# External Validation of Pre‐Treatment Primary Tumor Volume as a Prognostic Factor in Head and Neck Cancer Treated With (Chemo)Radiotherapy

**DOI:** 10.1002/hed.70315

**Published:** 2026-05-11

**Authors:** Gabriel Adrian, Andre Haraldsson Änghede, Lachlan McDowell, Maria Gebre‐Medhin

**Affiliations:** ^1^ Department of Hematology, Oncology, and Radiation Physics Skåne University Hospital Lund Sweden; ^2^ Department of Radiation Oncology Princess Alexandra Hospital Brisbane Australia; ^3^ Department of Clinical Sciences Lund, Division of Oncology Lund University Lund Sweden

**Keywords:** hypopharynx cancer, larynx cancer, oropharyngeal squamous cell carcinoma, prognostic factor, tumor volume, validation

## Abstract

**Background:**

Primary tumor volume (GTVp) is a key prognostic factor in oropharyngeal squamous cell carcinoma (OPSCC) treated with radiotherapy. We aimed to externally validate this association and assess its relevance in other head and neck sites.

**Methods:**

Patients from the RADCURE dataset were analyzed using Cox regression for local failure, progression‐free survival (PFS), and overall survival (OS). Subgroup analyses included HPV status, chemotherapy use, and separate evaluations for hypopharyngeal and laryngeal cancers. Model performance was assessed using the concordance index (C‐index).

**Results:**

Among 1286 OPSCC patients, increasing GTVp was associated with a higher risk of local failure and reduced PFS and OS, consistent across HPV and chemotherapy subgroups. Similar associations were observed in hypopharyngeal (*n* = 157) and laryngeal (*n* = 803) cancers. Model performance was strong (C‐index up to 0.82).

**Conclusion:**

Primary tumor volume is a robust prognostic factor across head and neck cancer sites, supporting risk stratification in curative radiotherapy.

## 1 Introduction

Radiotherapy (RT) is the primary treatment option for many head and neck squamous cell carcinoma (HNSCC) patients. Daily treatment for several weeks gradually kills of the tumor cells, and to achieve cure, all tumor cells must be eradicated. The starting point for this battle, that is, the number of cells the irradiation must sterilize, has been known for decades to influence the outcome [1]. Congruently, in a clinical context, the tumor volume has been investigated as a prognosticator. Although some studies did not establish an association between tumor volume and outcome in HNSCC [2, 3], the majority of the studies have [4, 5, 6, 7, 8, 9, 10, 11, 12, 13, 14, 15, 16, 17]. However, analyses differ across studies, with the tumor volume entity defined as either primary tumor volume, or nodal volumes, or both, as well as varying endpoints (e.g., local, regional or distant failure, progression free survival, overall survival). In addition, statistical methods differ, with tumor volume incorporated as a dichotomized (e.g., large vs. small, at a random or pre‐defined cut‐off) or as a continuous variable (often per cm^3^ or 10 cm^3^‐increase, or the logarithm of the volume). These variations across studies impose challenges for validation of specific findings.

We recently investigated outcomes for patients with oropharyngeal squamous cell carcinoma (OPSCC) and showed that primary tumor volume had a major role for prognostication, including local failure, progression‐free survival and overall survival [13]. In the current short communication, we present the validation of these findings in an independent cohort. The findings were also validated for hypopharyngeal and laryngeal cancers.

## 2 Methods

The RADCURE data set, publicly available through The Cancer Imaging Archive (TCIA), was used [18, 19]. Baseline clinical features included age, sex, human papillomavirus (HPV)‐status (for OPSCC), performance status, smoking history, T‐ and N‐staging, as well as radiotherapy details (total irradiation dose and number of fractions). Only patients with squamous cell carcinoma histology were included. Patients denoted as “post‐operative RT” were excluded from the analyses. For the oropharynx cohort, doses corresponding to an estimated equivalent dose in 2 Gy fractions (EQ D2) of ≥ 68 Gy corrected for assumed overall‐treatment time were included. The primary tumor volume (GTVp) was available in the accompanying CT dataset, from which the volume of GTVp was extracted.

All time‐to‐event endpoints were calculated from start of radiotherapy. Follow‐up time was estimated using the inverse Kaplan–Meier method. Univariable and multivariable Cox‐regression analyses were used for effect estimates. The GTVp was included as a continuous variable, yielding hazard ratio [HR] per cm^3^ increase in volume. Schönfeld residuals were used to assess the validity of the proportional hazard assumption. To illustrate the impact of GTVp, Kaplan–Meier were used with patients grouped into “tumor volume doublings”, that is, each consecutive group consisted of patients with tumor twice as large. Model performance was evaluated using the concordance index (C‐index), which measures the discriminative ability of the Cox‐regression models.

Baseline patient, tumor and treatment characteristics were compared between HPV‐positive and negative cases using chi‐square test for categorical variables, and Mann–Whitney for continuous variables. Because head and neck cancer comprises distinct disease entities, oropharyngeal, hypopharyngeal, and laryngeal cancers were analyzed separately.

For oropharyngeal tumors, additional subgroup analyses for HPV‐positive and HPV‐negative cases were conducted, as well as for patients treated with or without concurrent chemotherapy, in the HPV‐positive and HPV‐negative cohort. Tumor volume was also investigated within each T‐category. Exploratory analysis compared patients with small T4 (smaller than the median T4‐volume, 52 cm^3^) to large T2‐3 (larger than the median T4‐volume, 52 cm^3^). Additionally, the proportion of patients with failure within 3 years was investigated as a function of primary tumor volume. Sensitivity analyses were conducted by removing the patients with primary tumor volume larger than the 95th percentile, as well as including all patients, regardless of prescribed irradiation dose, and by analyzing those who received 70 Gy in 35 fractions. Exploratory analyses addressing total nodal volume (GTVn) were conducted.

All analyses and visualizations were made in R (version 4.2.2) using RStudio (version 2024.12.0 + 467) with the package survminer.

## 3 Results

### 3.1 Oropharynx Cohort

In the oropharynx cohort, 1286 cases were included (Table 1). Among these, 854 patients had HPV‐positive tumors, and 314 patients had HPV‐negative (not reported for 118). Median follow‐up time was 4.5 years (interquartile range [IQR] 2.6–6.2). One hundred eleven local failures, 466 PFS‐events, and 406 deaths occurred during follow‐up. Baseline patient, tumor, and treatment characteristics are shown in Table 1. The comparison between HPV‐positive and HPV‐negative cases underpins the different disease entities.

**TABLE 1:** Baseline patient and tumor characteristics in the oropharyngeal cohort, including separation by HPV‐status.

	All	HPV‐negative	HPV‐positive	*p*
	*n* = 1286	*n* = 314	*n* = 854	
Age, mean (SD)	61.5 (9.8)	64.4 (10.4)	60.5 (9.2)	< 0.001
Sex				
Female	243 (18.9)	84 (26.8)	140 (16.4)	< 0.001
Male	1043 (81.1)	230 (73.2)	714 (83.6)	
ECOG.PS				
ECOG 0	780 (60.7)	139 (44.3)	571 (66.9)	< 0.001
ECOG 1	397 (30.9)	130 (41.4)	232 (27.2)	
ECOG 2	88 (6.8)	33 (10.5)	46 (5.4)	
ECOG 3	15 (1.2)	10 (3.2)	4 (0.5)	
ECOG 4	2 (0.2)	1 (0.3)	0 (0.0)	
Unknown	4 (0.3)	1 (0.3)	1 (0.1)	
Smoking status				
Current	433 (33.7)	184 (58.6)	213 (24.9)	< 0.001
Ex‐smoker	517 (40.2)	110 (35.0)	354 (41.5)	
Non‐smoker	335 (26.0)	19 (6.1)	287 (33.6)	
Unknown	1 (0.1)	1 (0.3)	0 (0.0)	
Smoking pack‐year, mean (SD)				
T	24 (23)	38 (24)	18 (20)	< 0.001
T1	187 (14.5)	34 (10.8)	143 (16.7)	< 0.001
T2	435 (33.8)	89 (28.3)	309 (36.2)	
T3	384 (29.9)	94 (29.9)	245 (28.7)	
T4	280 (21.8)	97 (30.9)	157 (18.4)	
N				
N0	179 (13.9)	87 (27.7)	73 (8.5)	< 0.001
N1	120 (9.3)	42 (13.4)	65 (7.6)	
N2a	58 (4.5)	7 (2.2)	46 (5.4)	
N2b	499 (38.8)	81 (25.8)	378 (44.3)	
N2c	358 (27.8)	82 (26.1)	239 (28.0)	
N3	72 (5.6)	15 (4.8)	53 (6.2)	
M				
M0	1286 (100.0)	314 (100.0)	854 (100.0)	
Stage				
I	18 (1.4)	13 (4.1)	4 (0.5)	< 0.001
II	65 (5.1)	32 (10.2)	29 (3.4)	
III	157 (12.2)	54 (17.2)	82 (9.6)	
IVA	907 (70.5)	173 (55.1)	653 (76.5)	
IVB	138 (10.7)	42 (13.4)	85 (10.0)	
IVC	1 (0.1)	0 (0.0)	1 (0.1)	
HPV				
Negative	314 (24.4)	314 (100.0)	0 (0.0)	
Positive	854 (66.4)	0 (0.0)	854 (100.0)	
Not available	118 (9.2)			
Treatment modality				
ChemoRT	665 (51.7)	106 (33.8)	501 (58.7)	< 0.001
RT + EGFRi	45 (3.5)	13 (4.1)	30 (3.5)	
RT alone	576 (44.8)	195 (62.1)	323 (37.8)	
Fractionation schedule				
60 Gy/25 Fx	99 (7.7)	48 (15.3)	34 (4.0)	< 0.001
64 Gy/40 Fx	110 (8.6)	28 (8.9)	61 (7.1)	
68 Gy/34 Fx	5 (0.4)	0 (0.0)	4 (0.5)	
69.6 Gy/36 Fx	1 (0.1)	1 (0.3)	0 (0.0)	
70 Gy/35 Fx	1070 (83.2)	237 (75.5)	754 (88.3)	
74 Gy/37 Fx	1 (0.1)	0 (0.0)	1 (0.1)	
GTVp, median (IQR)	21.5 [10.8, 38.1]	24.2 [13.4, 43.4]	19.5 [9.6, 34.5]	< 0.001

Abbreviations: ChemoRT: concurrent chemotherapy; EGFRi: epidermal growth factor receptor‐inhibitor.

**FIGURE 1:**
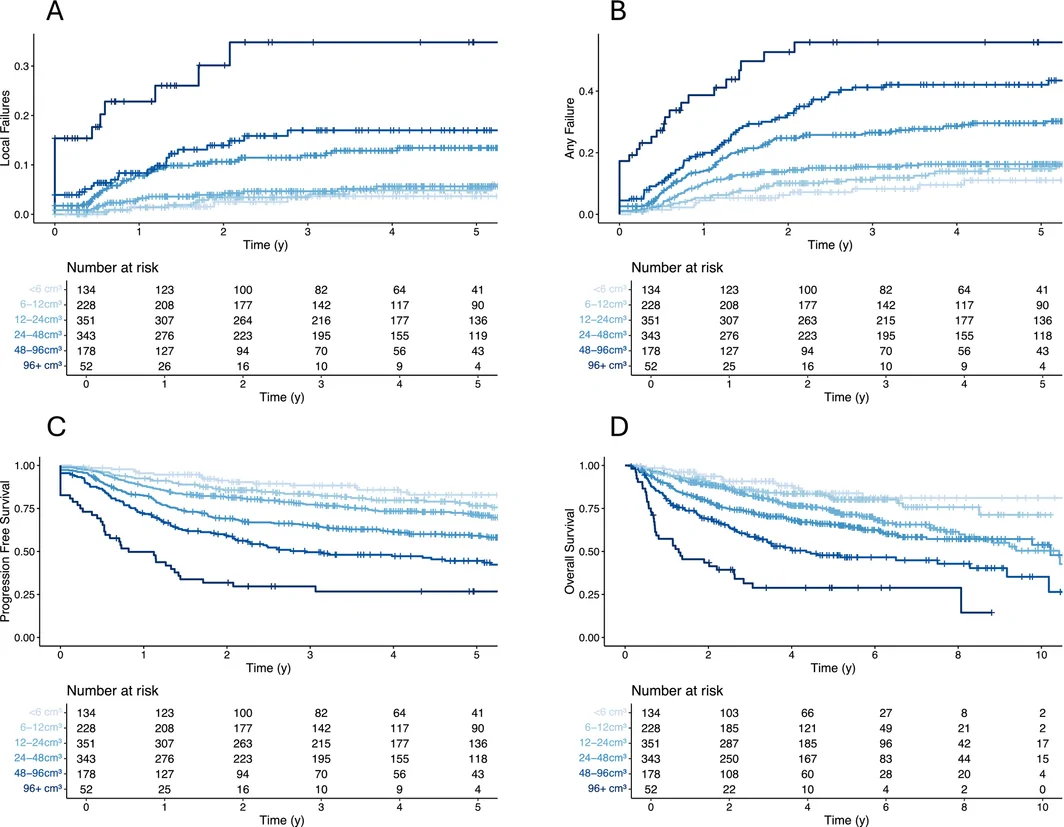
Patients with oropharyngeal squamous cell carcinoma, grouped into tumor volume doublings (< 6, 6–12, 12–24, 24–48, 48–96, and > 96 cm^3^) for local failure (A), any failure (B), progression‐free survival (C), and overall survival (D). [Color figure can be viewed at wileyonlinelibrary.com]

In the full cohort of OPSCC patients, increasing primary tumor volume was found to significantly increase the risk for local failure (HR 1.014 (95% CI 1.011–1.018) per cm^3^), progression free survival (HR 1.013 (95% CI 1.011–1.015) per cm^3^), and overall survival (HR 1.013 (95% CI 1.010–1.015) per cm^3^) in univariable analyses (Table 2 and illustrated in Figure 1). Adjusting for age, sex, performance status, smoking status, treatment modality (concurrent chemotherapy, concurrent EGFR‐inhibitors, or definitive RT alone), nodal classification, and HPV‐status in multivariable Cox‐regression analyses confirmed the association for primary tumor volume and outcome, with similar effect estimates as the univariable analyses (Table 2). In exploratory analyses, we investigated the inclusion of total nodal volume in addition to nodal classification in the multivariable models. The total nodal volume was borderline significant for the endpoint PFS (*p* = 0.051), but was not associated with the other studied endpoints, and it did not alter the effect estimates for primary tumor volume (Table S1).

**TABLE 2:** Cox‐regression analyzing the impact of increasing primary tumor volume (with HR per cm^3^) in univariable and multivariable models (adjusted for age, sex, performance status, N‐stage, HPV‐status, smoking status, use of concurrent chemotherapy or epidermal growth receptor‐inhibitor) for the three endpoints local failure, progression‐free survival (PFS) and overall survival (OS) in the oropharynx cohort.

Endpoint	Univariable			Multivariable		
	HR	CI	*p*	HR	CI	*p*
Local	1.014	1.011–1.018	< 0.001	1.016	1.012–1.02	< 0.001
PFS	1.013	1.011–1.015	< 0.001	1.013	1.010–1.015	< 0.001
OS	1.013	1.010–1.015	< 0.001	1.012	1.010–1.015	< 0.001

Abbreviations: CI: 95% confidence interval; HR: hazard ratio; Local: local failure; OS: overall survival; PFS: progression‐free survival.

When analyzing patients with HPV‐positive and HPV‐negative tumors separately, the impact of primary tumor volume remained significant for all three endpoints, as well as in subgroup analyses of patients treated with concurrent chemotherapy or definitive radiotherapy for both HPV‐positive and HPV‐negative cases (Table S2). Further, within each T‐category, primary tumor volume was associated with outcome, with the exemptions of all outcomes for T1 and local failures only for T2 (Table S2).

**FIGURE 2:**
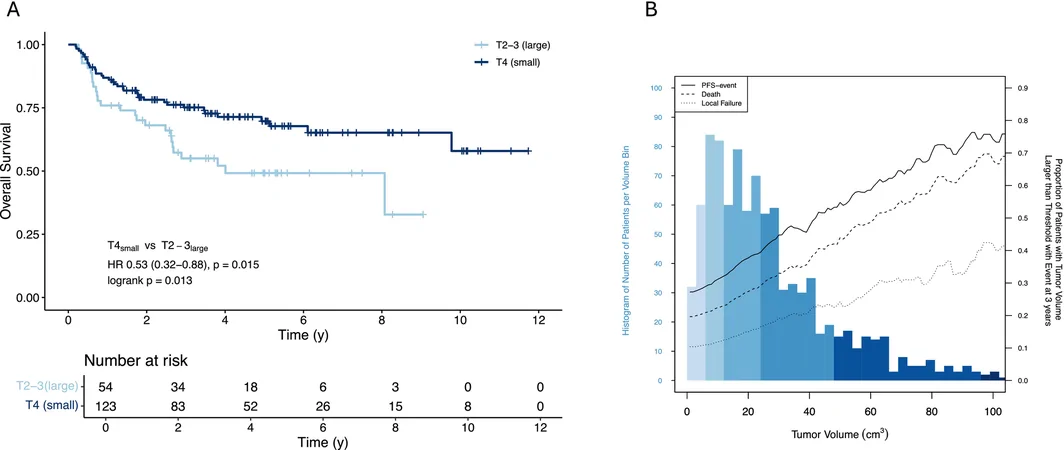
Patients with oropharyngeal squamous cell carcinoma comparing small T4‐tumors (volume less than median T4‐volume, 52 cm^3^) with large T2–T3‐tumors (volume larger than median T4‐volume, 52 cm^3^). Proportion of patients with event (local failure, PFS‐event or death) at 3 years as a function of primary tumor volume. [Color figure can be viewed at wileyonlinelibrary.com]

A considerable overlap between T‐category and primary tumor volume was found (Figure S1). Exploratory comparisons of patients with large T2 or T3 tumors (larger than the median T4‐volume, 52 cm^3^) compared with patients with small T4 (smaller than the median T4‐volume) revealed HR 0.53 (95% CI 0.32–0.88) for overall survival, indicating that tumor volume has a greater impact compared with T‐category in this particular setting (Figure 2A). Figure 2B illustrates the relationship between tumor volume and patients experiencing an event (local failure, PFS or OS) within 3 years.

Evaluation of the Cox‐regression models for local failures revealed excellent discrimination (C‐index 0.82 (95% CI 0.77–0.86)) and good discrimination for OS (C‐index 0.76 (95% CI 0.74–0.79)) when combining GTVp, HPV‐status and chemotherapy. The current validation set and the previous cohort [13] performed almost identically (Table S3).

### 3.2 Hypopharynx Cohort

One hundred fifty‐seven patients with squamous cell carcinoma in the hypopharynx were available for analysis. Baseline patient, tumor, and treatment characteristics are shown in Table S4. Twenty‐eight local failures, 111 PFS events, and 95 deaths were registered. Median follow‐up time was 4.3 years (IQR 1.8–6.2).

Primary tumor volume had a significant impact on the studied endpoints. The HR per cm^3^ increase in volume was 1.016 (95% CI 1.006–1.025) for local failure, HR 1.011 (95% CI 1.005–1.017) for PFS, and HR 1.013 (95% CI 1.007–1.019) for OS (Table S5 and Figure S2). The findings remained in multivariable analyses (Table S5).

### 3.3 Larynx Cohort

Eight hundred and three patients with laryngeal squamous cell carcinoma were analyzed. Baseline patient, tumor and treatment characteristics are shown in Table S4. One hundred twenty‐four local failures, 328 PFS events and 255 deaths were registered. Median follow‐up time was 3.9 years (IQR 2.2–5.5).

As expected for larynx tumors, the primary tumor volumes were significantly smaller compared with the oropharynx and hypopharynx cohorts. However, as for the other cohorts, primary tumor volume was associated with outcome. The HR per cm^3^ increase in volume was 1.021 (95% CI 1.013–1.029) for local failure, HR 1.022 (95% CI 1.017–1.026) for PFS, and HR 1.024 (95% CI 1.019–1.029) for OS (Table S6 and Figure S3). The findings remained in multivariable analyses (Table S6).

Similar to the oropharynx cohort, there was a substantial overlap between T‐category and primary tumor volume (Figure S1).

### 3.4 Sensitivity and Additional Analyses

To assess whether the largest tumors disproportionally influenced the results, sensitivity analyses were performed by excluding patients with tumor volume above the 95th percentile. These analyses generally yielded higher effect estimates (Table S7) suggesting that the observed association with primary tumor volume was not driven by outliers. On the contrary, when modeling GTVp as a continuous variable, the largest outliers may have attenuated the estimates.

Kaplan–Meier curves analyzing HPV‐positive and HPV‐negative OPSCC separately, as well as OPSCC treated with 70 Gy in 35 fractions, revealed similar results (Figure S7). Including all fractionation schedules did not alter the findings (Figure S5).

## 4 Discussion

In this independent dataset, we confirmed that primary tumor volume was highly associated with outcome in patients with oropharyngeal squamous cell carcinoma treated by RT. The finding was consistent across subgroups, including HPV‐positive and HPV‐negative cases, treated with concurrent chemotherapy or by RT alone. In addition, the findings also applied to hypopharyngeal and laryngeal primary tumors.

For squamous cell carcinoma of the head and neck, there is now considerable evidence that primary tumor volume is of major importance for outcome. Although the absolute effect size is not possible to compare across studies, since different endpoints and statistical approaches have been used, most studies present congruent findings [4, 5, 6, 7, 8, 9, 10, 11, 12, 13, 14, 15, 16, 17]. As discussed by others, variations across subsites of the head and neck probably influence volume‐analysis [20]. For the subsite oropharynx, stratification for HPV/p16‐status adds additional discrimination. Moreover, a mixture of patients receiving concurrent chemotherapy or definitive RT adds further challenges to compare results across studies. Despite these heterogeneities, only a few contradictory studies found that the effect of tumor volume was marginal [21, 22, 23, 24], or none [2, 3]. Our current findings agree well with our previous report [13]. In addition, the current analyses include separation for chemotherapy in addition to the HPV‐stratification, as well as congruent findings for larynx and hypopharynx tumors.

For most head and neck sites, T‐classification is defined by one‐dimensional tumor extent. However, almost mimicking our previous results [13], the distribution of primary tumor volume across T‐classification is considerable, both in the oropharynx and larynx. Our exploratory analysis also showed that primary tumor volume seems to triumph T‐category in certain scenarios (Figure 2A).

Of note, the present study does not incorporate sophisticated “radio‐omic” features or deep‐learning trained models [25]. Instead, its radiographic input is solely the volume of the delineated GTVp‐structure. When paired with HPV‐status and chemotherapy‐stratification, a C‐index > 0.80 is evident both in the present as well as the previous cohort (Table S2), establishing a baseline of these fundamental clinical parameters against which more advanced models can be compared. In our previous study, we suggested that primary tumor volume, in addition to its prognostic utility, appeared to be predictive for response to intensified treatment [13]. A similar analysis was not possible in the present cohort, since patients had not been allocated to random fractionation schedules, nor did it include information about overall treatment time. The benefit of hyperfractionated RT to 83.0 Gy in patients with large primary tumors is currently being investigated in the randomized ARTSCAN VI‐trial (trial number NCT06248996 at clinicaltrials.gov).

There are some limitations in the current study. A lack of details around overall treatment time prohibits a more detailed analysis of fractionation schedules. The analyses rely on the integrity of the open‐source data, TCIA. Additionally, the number of subjects in the hypopharyngeal cohort was small.

To conclude, the current findings validate that primary tumor volume is of major importance for outcome after RT in head and neck cancer. The results are consistent across endpoints, across subsites, and for OPSCC, in both HPV‐positive and HPV‐negative cases, regardless of treatment with concurrent chemotherapy or radical RT.

## Funding

This work was supported by Fru Berta Kamprads Stiftelse; Svenska Sällskapet för Medicinsk Forskning; Tegger Foundation.

## Ethics Statement

The authors have nothing to report.

## Consent

The authors have nothing to report.

## Conflicts of Interest

The authors declare no conflicts of interest.

## Supporting information


**FIGURE S1:** Comparison between T‐classification and primary tumor volume in the oropharynx and larynx cohorts.
**FIGURE S2:** Hypopharynx. Patients with hypopharyngeal squamous cell carcinoma, grouped into tumor volume doublings (< 12, 12–24, and > 24 cm3) for local failure (A), any failure (B), progression free survival (C), and overall survival (D).
**FIGURE S3:** Larynx. Patients with laryngeal squamous cell carcinoma, grouped into tumor volume doublings (< 1, 1–2, 2–4, 4–8, 8–16, and > 16 cm^3^) for local failure (A), any failure (B), progression free survival (C), and overall survival (D).
**FIGURE S4:** Oropharynx subgroup analyses.
**FIGURE S5:** Oropharynx, all fractionation schedules.
**TABLE S1:** Oropharynx cohort. Multivariable Cox‐regression analyzing the impact of increasing primary tumor volume (with HR per cm^3^) with or without inclusion of total nodal volume (GTVn) in addition to adjustment for age, sex, performance status, N‐classification, HPV‐status, smoking status, use of concurrent chemotherapy or epidermal growth receptor‐inhibitor. The table presents the effect estimates for the variables GTVp, N‐classification, and GTVn.
**TABLE S2:** Oropharynx cohort. Univariable Cox‐regression analyzing the impact of increasing primary tumor volume (with HR per cm^3^) across subgroups of oropharynx cancer patients.
**TABLE S3:** Oropharynx. Concordance Index for local failure and overall survival in the oropharynx cohort (validation cohort) and in the previous cohort [13].
**TABLE S4:** Baseline patient and tumor characteristics in the hypopharynx and larynx cohorts.
**TABLE S5:** Hypopharynx. Univariable and multivariable Cox‐regression analyzing the impact of increasing primary tumor volume (with HR per cm^3^). The multivariable analysis is adjusted for performance status and N‐stage (for local failure, only 28 events), and age, sex, performance status, N‐stage, HPV‐status, smoking status, use of concurrent chemotherapy or epidermal growth receptor‐inhibitor (for PFS and OS).
**TABLE S6:** Larynx. Univariable and multivariable Cox‐regression analyzing the impact of increasing primary tumor volume (with HR per cm^3^). The multivariable analysis is adjusted for age, sex, performance status, N‐stage, HPV‐status, smoking status, use of concurrent chemotherapy or epidermal growth receptor‐inhibitor.
**TABLE S7:** Oropharynx cohort, hypopharynx cohort, larynx cohort. Sensitivity analysis with large tumor (> above 95 percentile) removed. HR indicates the univariable analysis of GTVp (per cm^3^).

## Data Availability

The data that support the findings of this study are openly available in RADCURE at https://www.cancerimagingarchive.net/.
